# Prospective Evaluation of Individualized Nutrition Support on the Incidence of High-Output Ileostomy

**DOI:** 10.3390/nu18193301

**Published:** 2026-10-08

**Authors:** Rahel M. Strobel, Stella Toepfer, Konrad Neumann, Kati Engel, Michael Schumann, Wael Rayya, Christian H. W. Schineis, Katharina Beyer, Arved Weimann, Johannes C. Lauscher

**Affiliations:** 1 Department of General and Visceral Surgery, Charité—Universitätsmedizin Berlin, corporate member of Freie Universität Berlin und Humboldt-Universität zu Berlin, Campus Benjamin Franklin, Hindenburgdamm 30, 12203 Berlin, Germany; 2 Department for Urology, Franziskus Krankenhaus, 10787 Berlin, Germany; 3 Institute of Biometry and Clinical Epidemiology, Charité—Universitätsmedizin Berlin, corporate member of Freie Universität Berlin und Humboldt-Universität zu Berlin, 10117 Berlin, Germany; 4 Department of Gastroenterology, Infectiology and Rheumatology, Charité—Universitätsmedizin Berlin, corporate member of Freie Universität Berlin und Humboldt-Universität zu Berlin, 12203 Berlin, Germany; 5 Department for General, Visceral and Transplantation Surgery, University Medicine Augsburg, 86156 Augsburg, Germany; 6 Department of General, Visceral, and Oncological Surgery, St. George Hospital, 04129 Leipzig, Germany

**Keywords:** ileostomy, high-output stoma, clinical nutrition

## Abstract

Background: Patients with an ileostomy are at risk of developing a high-output ileostomy (HOS). An episode of HOS can lead to dehydration and acute kidney injury (AKI). Existing dietary recommendations are largely based on expert opinion. At Charité—Campus Benjamin Franklin, a new interdisciplinary nutrition concept was developed for patients following ileostomy creation. Methods: This prospective, single-centre interventional study included adult patients undergoing elective surgery with the creation of an ileostomy between July 2022 and November 2024. The primary outcome was the incidence of post-discharge HOS occurring during the follow-up period. Patients received standardized postoperative meals and individualized dietary counselling. Outcomes were compared to a historical cohort. Results: Among 103 patients (44.2% female; median age 47.5 years), HOS occurred in 13 patients (12.6%; 95% CI 6.8–20.4%) during follow-up and in 41 of 296 patients in the historical cohort (13.9%; 95% CI, 10.4–18.3%). Patients (*n* = 23) developing HOS during hospitalization experienced no recurrence at follow-up. Readmission was more frequent in the HOS group (53.8% vs. 21.1%, *p* = 0.011), particularly due to AKI (46.2% vs. 5.6%, *p* < 0.001). Weight loss was also more common during follow-up in the group with HOS (76.9% vs. 35.6%, *p* = 0.005), with a median weight loss of 2 kg. Conclusions: HOS incidence was not different from that observed in the historical cohort; however, the study was not designed to establish equivalence or non-inferiority. The structured nutrition pathway was feasible to implement. Post-discharge HOS was associated with readmission, acute kidney injury, and weight loss.

## 1. Introduction

In Germany, an estimated 32,000 people live with an ileostomy [1]. The most common indications for ileostomy creation are colorectal surgeries in patients with oncological diseases, inflammatory bowel diseases (IBDs) or familial adenomatous polyposis (FAP). Approximately 14–26% of patients with an ileostomy develop a high-output ileostomy (HOS) postoperatively [2,3]. The definition of HOS varies in the literature. Most authors refer to the cumulative stoma output over 24 h. HOS is defined in the literature as stoma output > 1500 mL on one day [4], >2000 mL on one day [5], >1000 mL per day for three consecutive days [2,6] or >2000 mL for three consecutive days [7]. In our study, HOS was defined as the loss of more than 1000 mL of liquid stool per day for three consecutive days. An episode of HOS can lead to dehydration and acute kidney injury (AKI). Approximately 20% of all patients with an ileostomy are rehospitalized postoperatively due to disturbances in fluid or electrolyte balance [8]. Evidence for nutritional advice is low, and existing dietary recommendations after ileostomy surgery are often only based on expert opinions [9].

At the Department of General and Visceral Surgery at the Benjamin Franklin Campus of Charité, an interdisciplinary team collaborated with the Department of Gastroenterology, Infectiology, and Rheumatology, the Department of Endocrinology, Diabetology, and Nutritional Medicine and the kitchen management to develop a new nutrition concept for patients after ileostomy surgery, based on current recommendations [10]. The primary objective of the NUTROMY study was to prospectively evaluate the feasibility of implementing a standardized nutrition concept with individualized dietary counselling and to describe the incidence of post-discharge HOS and related clinical outcomes.

## 2. Material and Methods

### 2.1. Study Design and Follow-Up

This pilot study was designed as a prospective, single-centre interventional study. Patients who were at least 18 years old and underwent an elective open, laparoscopic-assisted, or robot-assisted surgery to receive a terminal or double-barrel ileostomy for the first time were included. The ileostomy was usually constructed 20 to 30 cm proximal to the ileocecal valve. All patients were invited to attend a clinical and laboratory follow-up in the outpatient clinic approximately three months postoperatively. Alternatively, a telephone call was conducted to record stoma output volume.

### 2.2. Historical Control Group

Outcomes were compared with those of a historical control as a descriptive reference cohort rather. The historical cohort was conducted at the same surgical department of general and visceral surgery at the Benjamin Franklin Campus of Charité between 2012 and 2018. The retrospective cohort by Seifarth et al. included 296 patients and encompassed both emergency (33.8%) and elective surgeries [2]. The diagnosis for creation of ileostomy was ulcerative colitis in 33.4%, Crohn’s disease in 21.6% and colorectal carcinoma in 24.7%. Surgical procedures included left-sided colectomy (27.4%), proctocolectomy with ileo pouch-anal anastomosis (16.6%), proctocolectomy with end ileostomy (14.9%), right-sided colectomy (15.9%), ileostomy creation without bowel resection (17.6%) and small bowel resection (7.8%). HOS occurred in 41 of 296 patients (13.9%). This cohort was selected for comparison as it represented the standard of care for colorectal surgery in our department for general and visceral surgery. The patients’ and surgical characteristics were therefore comparable. A limitation of the comparison with the cohort described by Seifarth et al. is the lack of data regarding the exact onset of high-output ileostomy, which precluded the definition of a follow-up period.

### 2.3. Nutrition Concept—The NUTROMY Rationale

Each food item included in the nutrition plan was carefully selected by the interdisciplinary team. The diet emphasized low-fibre, non-osmotically active, well-tolerated and fresh foods with adequate protein and energy content. The postoperative diet was structured according to the patient’s postoperative day (see Figure 1). The dietary regimen adhered to the recommendations of the German Nutrition Society (Deutsche Gesellschaft für Ernährung, DGE). The principal focus of this diet was reduced fibre intake. The hospital diet kitchen structured its menu planning in accordance with the DGE reference values and quality standards for nutrient intake. The intended energy target was approximately 25 kcal/kg/day, and the protein target was approximately 1–1.2 g/kg/day. We supplemented with oral nutritional supplements whenever energy and protein targets were not met with the oral diet alone. During the early postoperative phase, fibre intake was restricted to approximately 10 g/day, with gradual progression according to tolerance. Patients were advised to achieve a total fluid intake of approximately 1500–2000 mL/day orally. We based our formulation on the World Health Organization (WHO) oral rehydration solution (ORS) [11]. The solution was prepared according to the WHO reduced-osmolarity oral rehydration formulation, containing per litre of water: 2.6 g sodium chloride, 13.5 g anhydrous glucose, 1.5 g potassium chloride, and 2.9 g trisodium citrate dihydrate. This provides 75 mmol/L sodium, 65 mmol/L chloride, 20 mmol/L potassium, 10 mmol/L citrate, and 75 mmol/L glucose, with a total osmolarity of 245 mOsm/L.

All study participants received at least 30 min of postoperative nutritional counselling delivered by one of two trained dietitians from the department of gastroenterology and endocrinology/diabetology/clinical nutrition during hospital stay. Counselling emphasized careful recording of fluid intake and stoma output, small frequent meals with the main meal at midday, and a low-fibre diet. Patients received preventive advice to drink oral fluids regularly throughout the day, avoid excessive intake of hypotonic fluids alone, and, where appropriate, separate fluid intake from meals. In cases of increased output, management included assessment of fluid balance, replacement of sodium and other electrolytes, administration of oral rehydration solutions, and intravenous fluids when oral replacement was insufficient.

Patients were advised to chew thoroughly, avoid gas-producing or highly fibrous fruits and vegetables, and were informed about possible lactose intolerance and its management. This was supported by a newly designed informational brochure on nutrition containing 16 pages. To assess the adherence to the recommendations from the individual dietary counselling, patients were interviewed in person or by telephone.

### 2.4. Outcomes

The primary outcome was the incidence of post-discharge high-output ileostomy (HOS) occurring during the follow-up period, defined as a stoma output exceeding 1000 mL of liquid stool per day for three consecutive days. During hospitalization, stoma output was measured in millilitres over consecutive 24 h periods using the ward’s standard collection and documentation procedures. The documented daily values were entered into the clinical record. After discharge, patients were instructed to measure output using a household measuring container and to record the values in the provided documentation materials or report them during follow-up. Acute kidney injury was defined according to the Kidney Disease: Improving Global Outcomes (KDIGO) criteria as any of the following: an increase in serum creatinine by ≥0.3 mg/dL within 48 h; an increase in serum creatinine to ≥1.5 times the baseline value, known or presumed to have occurred within the preceding 7 days; or urine output < 0.5 mL/kg/h for 6 h. AKI severity was classified as stage 1, 2, or 3 according to KDIGO criteria. Baseline serum creatinine was defined as the most recent preoperative value available before surgery. Urine output data were not systematically available during follow-up. Therefore, post-discharge AKI classification was based on serum creatinine criteria only.

In cases where HOS developed during the hospital stay, antidiarrheal therapy was initiated with pectin powder administered three times daily, followed by loperamide 2 mg starting with one dose in the morning and one dose in the evening. Depending on ileostomy output, the loperamide dose was gradually increased up to a maximum of eight 2 mg tablets per day. Persistent HOS despite this regimen prompted escalation of antidiarrheal treatment with opium tincture. Secondary outcomes were assessed during hospitalization and three months postoperatively. HOS onset was classified as occurring during hospitalization or after discharge.

### 2.5. Statistical Analysis

The primary endpoint (HOS during follow-up) was evaluated using a two-sample comparison with confidence intervals and reporting the absolute risk difference with an appropriate 95% confidence interval only as an exploratory comparison. Because this was a pilot feasibility study, no formal sample-size calculation for efficacy or non-inferiority was performed. Secondary outcomes were analyzed descriptively and considered exploratory. No formal adjustment for multiplicity was applied because the study was designed as a pilot feasibility evaluation rather than a confirmatory hypothesis-testing trial. *p*-values for secondary outcomes should therefore be interpreted cautiously. The secondary endpoints were described using absolute and relative frequencies and compared between groups using the *t*-test for independent variables when normal distribution was present and the Mann–Whitney U test for metric variables with skewed distribution. Fisher’s exact test was used in variables with small expected cell counts or zero cells. Analyses were performed using complete-case analysis for each outcome. No imputation was performed because this was an exploratory pilot study with a limited sample size and missing data across a few secondary outcomes. The number of missing observations is reported in the corresponding tables. Associations between categorical variables were examined through cross tabulation and chi-square test. The study was designed as a pilot study for preliminary assessment and feasibility evaluation. Statistical analysis was performed using IBM SPSS Statistics 30^®^ (IBM, Armonk, NY, USA).

### 2.6. Ethics

The ethics committee of Charité—University Medicine Berlin (application number EA4/078/22 on 10 June 2022) approved the study. Patient enrollment began on 22 July 2022. The NUTROMY study was registered at Deutsches Register Klinischer Studien (German Clinical Trials Register) with the code DRKS00030573 on 25 October 2022. The trial was conducted in accordance with the ethical principles of the Declaration of Helsinki and the principles of Good Clinical Practice (ICH-GCP E6) [12]. Written informed consent was obtained from all study participants. A comprehensive data protection concept was planned and accepted by the Clinical Trial Office of Charité—University Medicine Berlin in February 2022. The content of the article has been written in accordance with the STROBE checklist.

## 3. Results

### 3.1. Baseline Characteristics

A total of 104 patients were enrolled in the study between 22 July 2022 and 4 November 2024. The cohort comprised 46 females (44.2%) and 58 males (55.8%), with a median age of 47.5 years (IQR: 33–61.5 years). Nearly 60% of participants suffered from inflammatory bowel disease, and 38.5% were receiving immunosuppressive therapy. Other indications for ileostomy formation—categorized as “varia”—included perianal fistula, FAP, and protective ileostomy following bowel continuity restoration. The most performed surgical procedure was colectomy, including segmental resections such as right- or left-sided hemicolectomy, accounting for 35.6% of cases. Baseline characteristics of patients with and without HOS were summarized in Table 1.

### 3.2. Rate of Post-Discharge High-Output Ileostomy (HOS)

Follow-up was completed by 103 patients; one patient died during the hospital stay. Post-discharge HOS occurred in 13 of 103 patients (12.6%; 95% CI, 6.8–20.4%) during the prospective follow-up period. In the historical cohort, HOS was reported in 41 of 296 patients (13.9%; 95% CI, 10.4–18.3%) [2]. The absolute risk difference between the two cohorts was 1.2% percentage points (95% CI, −6.3 to 8.8%), calculated using the normal approximation for the difference between two proportions. However, the historical dataset did not provide sufficient information regarding the exact timing of HOS onset or a standardized follow-up window. Therefore, these rates should not be interpreted as a direct comparison of identical time-specific endpoints.

### 3.3. Risk Factors for Any Postoperative HOS

In univariate analysis a significant difference in age was observed between patients who developed HOS and those who did not. Patients with HOS were older, with a median age of 55.5 years compared to 45.0 years in patients without HOS (*p* = 0.027). Additionally, arterial hypertension was more prevalent among patients with HOS, affecting 12 patients (33.3%) versus 11 patients (16.2%) without HOS (*p* = 0.045) (see Table 1). The multivariable logistic regression model included all variables that were statistically significant in univariate analysis (age and arterial hypertension), as well as additional parameters considered to be of clinical relevance. Furthermore, logistic regression analysis did not identify any independent risk factors for HOS within this cohort (see Table 2).

### 3.4. Effects of HOS During Hospital Stay

The incidence of HOS during hospital stay was 22.1%, occurring in 23 of 104 patients (initially enrolled in the trial). The median time to onset of HOS was postoperative day 5 (IQR: 3–7 days). Both the volume and duration of parenteral fluid administration were significantly greater in patients with HOS (*p* = 0.010 and *p* = 0.001, respectively). Overall, 14 of 104 patients (13.5%) required supplementary parenteral nutrition—26.1% with HOS compared to 9.9% without HOS (*p* = 0.044). Acute kidney injury was rare and occurred in one patient with HOS (4.3%) and five patients without HOS (6.2%) (*p* = 0.740). Dialysis was not required at all. Electrolyte levels—including sodium, potassium, magnesium, calcium, and phosphorus—showed no significant differences prior to discharge. Hospital length of stay was significantly prolonged in patients who developed HOS with a median duration of 15 days compared to 10 days in patients without HOS (*p* = 0.002). Notably, five patients (21.7%) with HOS developed additionally postoperative paralytic ileus (see Table 3).

### 3.5. Occurrence of Complications Related to HOS During Follow-Up (Post-Discharge HOS)

Follow-up was completed by 103 patients; one patient died during the hospital stay. Follow-up of 11 patients was assessed by telephone only. During follow-up, HOS was observed in 13 of 103 patients (12.6%). The median postoperative day at which HOS occurred was 37 (IQR: 26.5–59). In all patients who experienced HOS during their hospital stay, ileostomy output had normalized by the time of follow-up. All 13 patients with HOS at follow-up developed the condition de novo after hospital discharge. The Kaplan–Meier curve illustrates the probability of remaining free of post-discharge HOS over time, with censoring indicated for patients whose follow-up ended without an HOS event. The analysis demonstrates that HOS events occurred over the observed follow-up period rather than being restricted to a predefined 90-day window. By postoperative day 90, 3 of 103 patients (2.9%) had developed a HOS (see Figure 2).

In univariate analysis patients with HOS were significantly older, with a median age of 62 years compared to 46 years in those without HOS (*p* = 0.003). No association was found between the underlying indication for surgery and the occurrence of HOS during follow-up. Hospital readmission was more frequent among patients with HOS (53.8%)—including five patients (38.5%) readmitted for acute kidney injury—compared to 21.1% of patients without HOS (*p* = 0.011). Acute kidney injury was significantly more common in the HOS group during follow-up (46.2% vs. 5.6%; *p* < 0.001). Moreover, weight loss was significantly more frequent in patients with HOS, affecting 10 patients (76.9%) with HOS compared to 32 patients (35.6%) without HOS (*p* = 0.005), with a median weight loss of 2 kg (see Table 4).

## 4. Discussion

This prospective, single-centre study included 104 patients undergoing elective surgery with the creation of an ileostomy. It is a prospective evaluation of a newly implemented nutrition concept focusing on low-fibre, fresh foods providing adequate protein and caloric intake in hospital combined with individualized nutritional counselling. It provides data on the feasibility of the implementation of the nutrition concept and on the incidence and clinical correlates of HOS in a prospectively followed cohort. Notably, patients who developed HOS during initial postoperative hospitalization who received tailored dietary and medical counselling, including antidiarrheal therapy, experienced no HOS recurrence at follow-up. The incidence of HOS was more frequent during initial hospitalization (*n* = 23, 22.1%) than during follow-up after hospital discharge (*n* = 13, 12.6%). The 13 patients (12.6%) with HOS at follow-up developed the condition de novo after hospital discharge. The comparison with the historical cohort [2] should be interpreted cautiously. The prospective cohort included elective procedures only, whereas the historical cohort included 33.8% emergency procedures and differed in the distribution of diagnoses and surgical procedures. However, the nutrition concept may facilitate the development of a clinical pathway by addressing the current lack of standardized care and dietary education for patients with ileostomy, especially when combined with individualized counselling and supported by a newly designed informational brochure on nutrition. In a separate publication based on patient-reported outcomes, adherence to dietary recommendations was associated with fewer HOS events; however, adherence was not prospectively quantified or validated in the present study, and this finding should not be interpreted as evidence of a causal preventive effect in the current cohort [13]. An umbrella review analyzing 22 systematic reviews identified only two that focused specifically on HOS as a complication of ileostomy creation [14]. In the NUTROMY trial, the length of hospital stay was prolonged in patients who developed HOS during hospitalization, with a median extension of 5 days. At follow-up, patients with HOS had higher rates of hospital readmission, particularly for acute kidney injury.

The incidence of HOS during follow-up in our study was 12.6%, placing it at the lower end of the range reported in the literature, which varied between 13% and 24% [2,7,15,16] (see Table 5). Reported rates varied depending on the postoperative interval used to define HOS. In our study, a three-month postoperative timeframe was chosen, as this corresponds to the typical clinical evaluation for potential ileostomy reversal. Baker et al. recorded a 16% incidence of HOS among 456 ileostomy patients before discharge and within the first three weeks postoperatively [7]. It is essential to recognize the occurrence of HOS by measuring daily stoma output—not only during hospitalization but also by educating patients to continue monitoring output after discharge. The greater use of antidiarrheal medication and parenteral fluids among patients with HOS reflects treatment escalation in response to increased stoma output and should not be interpreted as an independent risk factor or adverse consequence. Acute kidney injury was rare and not significantly different between groups, and no electrolyte abnormalities were noted. Mechanical ileus and paralytic ileus were more frequent among patients with in-hospital HOS. However, the temporal sequence of these events was not consistently recorded. Therefore, the present data cannot determine whether these complications preceded HOS, resulted from HOS-related fluid and electrolyte disturbances, or reflected shared underlying postoperative processes. Literature supports a bidirectional interaction [17]: HOS may precipitate other complications such as paralytic ileus through mechanisms like dehydration and electrolyte imbalance, which impair gastrointestinal motility. Conversely, the presence of other complications—such as intraabdominal infections—can act as risk factors for HOS development [18].

In our study, patients who developed HOS during follow-up experienced significantly higher rates of acute kidney injury, which frequently necessitated hospital readmission. Fielding et al. demonstrated in a retrospective analysis of 1213 patients that the creation of an ileostomy increases the risk of postoperative renal impairment due to dehydration [19]. More than half of the patients in our trial with HOS during follow-up were readmitted to hospital. In a retrospective study, unplanned readmission occurred in 28% of patients within 60 days following ileostomy formation. Dehydration was identified as the cause in 42% of these cases, occasionally resulting in acute kidney injury, significant electrolyte imbalances, or cardiac arrhythmias [20]. Patient education on monitoring stoma output and maintaining fluid balance remains crucial and must be continuously reinforced to prevent complications. Impairment of nutritional status was evident in patients with HOS. These patients experienced greater weight loss during follow-up compared to those without HOS, with a median weight loss of 2 kg. Consistent with a recent publication, 51.9% of patients fulfilled the GLIM criteria for malnutrition. Preoperative malnutrition by GLIM was not associated with HOS [13]. At follow-up, 25 of 103 patients (24.3%) had a MUST score of ≥2. Because nutritional status was assessed using a screening tool rather than a comprehensive diagnostic framework, these data should be interpreted as an indication of malnutrition risk rather than a diagnosis of malnutrition. The negative impact of an ileostomy on nutritional status was confirmed by other trials [21,22]. In a pilot study by Vasilopoulos et al. involving 13 patients with ileostomy and colorectal cancer, patients exhibited significant weight loss, lower body mass index, and reduced mid-arm circumference 20 days postoperatively [22]. For patients with an ileostomy, regular monitoring of weight and food intake postoperatively is essential. One study evaluating the dietary habits of 17 ileostomy patients reported inadequate intake of carbohydrates (58.8%), fats (88.2%), potassium (94.1%), magnesium (76.5%), vitamin B12 (64.7%), and overall energy (58.8%) [23]. In the early postoperative period, kidney function should be closely monitored by assessing serum urea, creatinine, and electrolytes [24].

Existing dietary recommendations after ileostomy surgery are often only based on expert opinions [9]. Mitchell et al. conclude in their scoping review that dietetic input should focus on personalized counselling, monitoring hydration and micronutrient risks. People with an ileostomy should initially follow a low-fibre, low-residue diet, gradually reintroducing a varied, higher-fibre intake as tolerated while monitoring for obstruction, high output and symptoms. Adequate fluid and sodium intake is essential to prevent dehydration, with many sources recommending at least 1.5–2 L/day plus extra salt, adjusted according to stoma output. Patients are advised to eat regularly (often small, frequent meals), chew thoroughly, and individualize food avoidance, only excluding items that repeatedly cause unacceptable symptoms such as bloating, high output, or blockage. In our study, each component of the newly implemented nutrition concept was carefully selected by an interdisciplinary team, with a focus on low-fibre, fresh foods providing adequate protein and caloric intake. This aligns with the recent dietary recommendations by Wang et al., who advise that patients with ileostomies should avoid high-fibre foods such as oats, corn, and legumes, and limit intake of foods high in simple sugars, fats, and sugary beverages [24]. The dietary approach in the NUTROMY trial was complemented by individualized nutritional counselling for each patient during the postoperative course. Notably, among patients who developed HOS during initial hospitalization, those who received structured dietary and medical counselling including antidiarrheal therapy did not experience a recurrence of HOS during follow-up. No significant electrolyte disturbances were observed in association with HOS during follow-up. In another trial, hypomagnesaemia was observed in 33% of patients who developed HOS three weeks postoperatively [25]. Mitchell et al. conducted an online survey involving 291 stoma patients to assess the availability and quality of nutritional counselling following stoma formation. The study revealed that not all patients who wished to receive dietary guidance were provided with it. However, among those who did receive counselling, 89.1% reported making dietary changes, and more than half found these changes helpful in managing their ileostomy [26]. Our trial, together with the aforementioned studies, highlights the importance of individualized dietary counselling for patients with an ileostomy.

Several potential limitations of the study need to be considered. First, this study was limited by its single-centre design, which may restrict the generalizability of the findings to other clinical settings or populations. The patient cohort reflected the practices, expertise, and demographic characteristics specific to our institution, potentially limiting external validity. Future multicenter studies with larger populations are warranted to confirm and extend these results. Second, our definition of HOS—as an ileostomy output exceeding 1000 mL of liquid stool per day for three consecutive days within a three-month postoperative period—differed from definitions and time frames used in other studies. The timing definition of HOS significantly influences study outcomes and comparability. Studies that define HOS onset strictly during the initial hospital stay tend to report lower overall incidence rates. Third, patients received the standardized meals provided according to the menu for ileostomy only during their inpatient stay and not after discharge. Moreover, adherence to the prescribed diet post-discharge was not monitored, and it is likely that not all patients strictly followed the nutrition concept, which may have attenuated its overall impact. Thus, there is an absence of systematic monitoring or verification of adherence to the prescribed nutritional regimen. Adherence was only assessed by an unvalidated, structured interview based on the recommendations provided during the inpatient counselling session. Fourth, the historical cohort for comparison of our HOS rate was retrospective and more heterogeneous, limiting the comparability of results. There was no comprehensive follow-up for the detection of HOS, thus an underestimation of the rate of HOS is possible in the historical cohort. The historical comparison is descriptive rather than causal. Still, our study provides prospective data from a well-defined and well-documented cohort with excellent nutritional counselling showing that no patient with HOS during hospital stay developed a recurrence of high-output ileostomy during the three-month follow-up.

**Table 5:** Overview of recent studies on HOS.

Authors	Year	Study Type	Patients (*n*)	Key Findings
Seifarth et al. [2]	2021	Retrospective cohort study	296	HOS incidence: 13.9% (definition: >1000 mL/day for ≥3 days). Independent risk factors: older age, Crohn’s disease (OR 11.33), right-sided colectomy/ileopouch anal anastomosis, ileostomy alone, small bowel resection.
Lee et al. [27]	2021	Retrospective cohort study	302	HOS incidence: 15.9% (≥2000 mL/day). HOS associated with postoperative ileus (POI) (45.8% vs. 7.5%, *p* < 0.001). Readmission rate: 16.9%. POI independent risk factor for readmission (OR 2.64) and ileus-related readmission (OR 3.87).
Stoop et al. [28]	2024	Systematic review (pediatric population)	Multiple studies (pediatric HOS)	Objective: Clarify evidence-based therapeutic approaches for high-output enterostomy in children. Highlights limited data and heterogeneous management strategies in pediatrics.
Baker et al. [7]	2011	Retrospective cohort study	687 stomas (456 ileostomies/jejunostomies)	Early HOS in 16% of ileostomies/jejunostomies. Most common causes: jejunostomy (<200 cm proximal small bowel remaining) and intra-abdominal sepsis/obstruction; 71% could be weaned from parenteral fluids; 61% had spontaneous resolution.
Hara et al. [4]	2020	Retrospective cohort study	164 (colorectal cancer)	HOS incidence: 31%. Organ/space infection frequently associated with HOS and outlet obstruction in defunctioning ileostomies.
Liu et al. [29]	2021	Systematic review and meta-analysis	Not applicable; pooled analysis of available studies	Eight factors were significantly associated with dehydration-related readmission after ileostomy formation: age ≥ 65 years, BMI ≥ 30 kg/m^2^, diabetes mellitus, arterial hypertension, renal comorbidity, regular diuretic use, ileal pouch–anal anastomosis, and longer length of stay after the index admission. A preoperative diagnosis of colorectal cancer was associated with a lower risk of dehydration-related readmission.
Vogel et al. [30]	2022	Systematic review and meta-analysis	82,451 patients from 71 studies	The pooled 30-day dehydration-related readmission rate after ileostomy creation was 6% (95% CI, 4–9%), while the pooled all-cause readmission rate was 20% (95% CI, 18–23%). The study highlighted substantial variability in reported readmission rates and the need for standardized definitions and prevention strategies.
Ozata et el. [31]	2024	Prospective prevention-bundle study; comparative before-and-after study	104	A prospective ileostomy-induced dehydration prevention bundle including structured patient education, post-discharge monitoring, and stepwise clinical management was associated with a reduction in dehydration-related readmissions from 22% in the pre-bundle group to 2% in the bundle group (p=0.002).

## 5. Conclusions

This prospective, single-centre pilot study evaluated the feasibility of implementing a structured inpatient nutrition pathway with individualized dietary counselling in patients undergoing elective ileostomy creation. The incidence of post-discharge HOS (12.6%) was not statistically different from that observed in a heterogeneous historical cohort, although the study was not designed to establish equivalence or a causal treatment effect. Notably, patients developing HOS during initial postoperative hospitalization who received tailored dietary and medical counselling including antidiarrheal therapy experienced no HOS recurrence at follow-up. HOS was associated with readmission, acute kidney injury, and weight loss. These findings support the feasibility of standardized nutritional counselling and provide a basis for a controlled, adequately powered prospective trial.

## Figures and Tables

**Figure 1:**
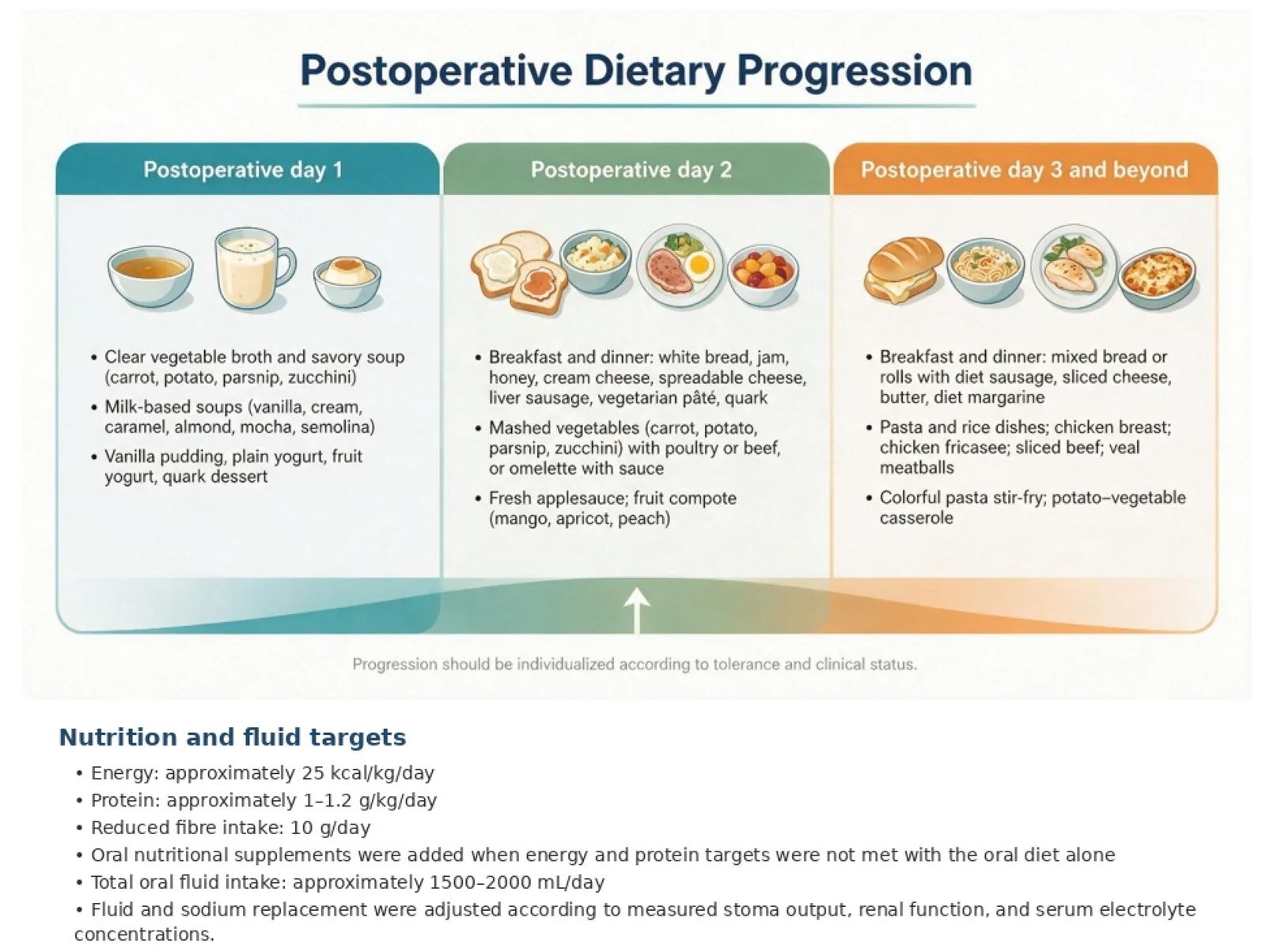
Postoperative dietary progression in hospital. During the preparation of this work, the authors used Perplexity (Perplexity AI, August 2026 version) to assist with this figure. After using this tool, the authors reviewed and edited the content as needed and take full responsibility for the content of the published article.

**Figure 2:**
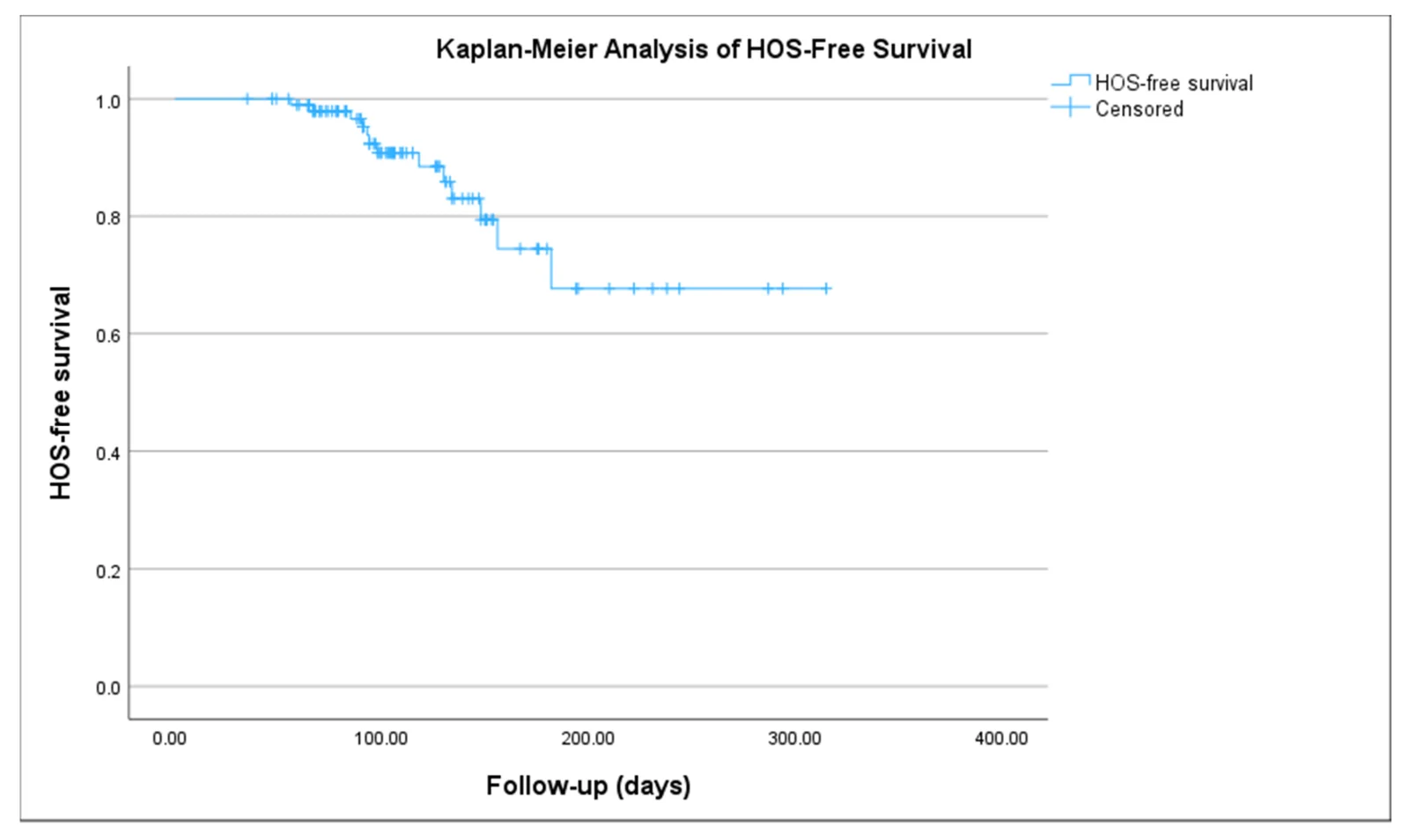
Kaplan–Meier time-to-event analysis of post-discharge HOS.

**Table 1:** Baseline characteristics of study participants. The occurrence of HOS was recorded if it took place either during the hospital stay or in the follow-up period (defined as any postoperative HOS).

	HOS (*n* = 36)	No HOS (*n* = 68)	Total (*n* = 104)	*p* Value
Sex				0.202 §
Female	19 (52.8%)	27 (39.7%)	46 (44.2%)	
Male	17 (47.2%)	41 (60.3%)	58 (55.8%)	
Age (years) (median; IQR)	55.5 (40.3; 63)	45.0 (28.3; 59)	47.5 (33; 61.5)	0.027 *†
BMI (kg/m^2^)	23.9 (20.8; 27.4)	23.8 (19.9; 27.1)	23.9 (20.3; 27.2)	0.774 †
BMI				0.218 §
Underweight	4 (11.1%)	10 (14.7%)	14 (13.5%)	
Normal weight	19 (52.8%)	27 (39.7%)	46 (44.2%)	
Overweight	8 (22.2%)	23 (33.8%)	31 (29.8%)	
Obesity I	0 (0%)	4 (5.9%)	4 (3.8%)	
Obesity II	4 (11.1%)	4 (5.9%)	8 (7.7%)	
Obesity III	1 (2.8%)	0 (0%)	1 (1.0%)	
ASA				0.916 §
1	3 (8.3%)	6 (8.8%)	9 (8.7%)	
2	26 (72.2%)	51 (75.0%)	77 (74.0%)	
3	7 (19.4%)	11 (16.2%)	18 (17.3%)	
Underlying diagnosis				0.648 §
Crohn’s disease	7 (19.4%)	22 (32.4%)	29 (27.9%)	
Ulcerative colitis	8 (22.2%)	15 (22.1%)	23 (22.1%)	
Ulcerative colitis-associated colorectal carcinoma	4 (11.1%)	5 (7.4%)	9 (8.7%)	
Colorectal carcinoma	12 (33.3%)	20 (29.4%)	32 (30.8%)	
Varia	5 (13.9%)	6 (8.8%)	11 (10.6%)	
Type of surgery				0.460 §
Ileostomy alone	5 (13.9%)	13 (19.1%)	18 (17.3%)	
Colectomy	16 (44.4%)	21 (30.9%)	37 (35.6%)	
Rectal resection	9 (25.0%)	15 (22.1%)	24 (23.1%)	
Proctocolectomy with ileo-pouch-anal anastomosis	2 (5.6%)	4 (5.9%)	6 (5.8%)	
Ileocecal resection	1 (2.8%)	8 (11.8%)	9 (8.7%)	
Segmental resection of colon or ileum	0 (0%)	3 (4.4%)	3 (2.9%)	
Varia	3 (8.3%)	4 (5.9%)	7 (6.7%)	
Comorbidities				
Coronary artery disease	1 (2.8%)	4 (5.9%)	5 (4.8%)	0.481 §
Arterial hypertension	12 (33.3%)	11 (16.2%)	23 (22.1%)	0.045 *§
Diabetes mellitus	3 (8.3%)	2 (2.9%)	5 (4.8%)	0.221 §
Nicotine abuse	8 (22.2%)	10 (14.7%)	18 (17.3%)	0.335 §
Elevated serum creatinine	7 (19.4%)	10 (14.7%)	17 (16.3%)	0.556 §
Immunosuppressive medication	12 (33.3%)	28 (41.2%)	40 (38.5%)	0.434 §

This table shows the initial 104 enrolled patients. Data were *n* (%) or median (25%; 75% interquartile range), §, chi-square test; * *p* ≤ 0.05; †, Mann–Whitney U test; HOS, high-output ileostomy; BMI, body mass index; underweight, BMI < 18.5 kg/m^2^; normal weight, BMI 18.5–24.9 kg/m^2^; overweight, BMI 25–29.9 kg/m^2^; obesity I, BMI 30.0–34.9 kg/m^2^; obesity II, BMI 35.0–39.9 kg/m^2^; obesity III, BMI > 40 kg/m^2^; ASA, American Society of Anesthesiology; elevated creatinine, >0.9 mg/dL in females and >1.2 mg/dL in males.

**Table 2:** Logistic regression of potential risk factors for HOS.

Independent Variables	OR (95% CI)	*p* Value
Age	1.02 (0.99–1.06)	0.229
ASA score	0.76 (0.31–1.91)	0.564
BMI	0.99 (0.91–1.07)	0.751
Arterial hypertension	2.18 (0.64–7.40)	0.211
Crohn’s disease vs. ulcerative colitis	1.59 (0.46–5.55)	0.465
Crohn’s disease vs. colorectal carcinoma	0.97 (0.26–3.60)	0.961
Crohn’s disease vs. ulcerative colitis-associated colorectal carcinoma	2.16 (0.42–11.19)	0.358
Crohn’s disease vs. varia	2.57 (0.52–12.65)	0.247

HOS, high-output ileostomy; OR, odds ratio; CI, confidence interval; ASA, American Society of Anesthesiology; BMI, body mass index; varia included perianal fistula, FAP, and protective ileostomy following bowel continuity restoration.

**Table 3:** Characteristics of patients with HOS during hospital stay (defined as in-hospital HOS).

	HOS (*n* = 23)	No HOS (*n* = 81)	Total (*n* = 104)	Missing	*p* Value
Need for antidiarrheal medication	21 (91.3%)	10 (12.3%)	31 (29.8%)	-	<0.001 *§
Quantity of parenteral fluid per day in millilitres (median; IQR)	1692 (1059; 2000)	1125 (1000; 1621)	1250 (1000; 1750)	16	0.010 *†
Duration of administration of parenteral fluids (days) (median; IQR)	6 (4; 13)	4 (3; 6)	4 (3; 7)	15	0.001 *†
Need for parenteral nutrition	6 (26.1%)	8 (9.9%)	14 (13.5%)	-	0.044 *§
Length of parenteral nutrition (days) (median; IQR)	6 (5.3; 16)	6 (3.5; 8.0)	6 (4.5; 8.8)	90	0.551 †
Serum creatinine (mg/dL) (median; IQR)	0.70 (0.64; 0.89)	0.76 (0.61; 0.93)	0.76 (0.62; 0.91)	-	0.934 †
Acute kidney injury	1 (4.3%)	5 (6.2%)	6 (5.8%)	-	0.740 §
Albumin in g/L (median; IQR)	33.8 (32.8; 37.5)	33.7 (28.6; 37.3)	33.8 (29.6; 37.4)	14	0.371 †
Magnesium in mmol/L (median; IQR)	0.80 (0.73; 0.90)	0.80 (0.74; 0.85)	0.80 (0.74; 0.85)	18	0.769 †
Calcium corrected in mmol/L (median; IQR)	2.41 (2.34; 2.51)	2.40 (2.33; 2.48)	2.40 (2.33; 2.48)	17	0.667 †
Sodium in mmol/L (median; IQR)	137 (134; 140)	139 (137; 140)	139 (137; 140)	-	0.050 †
Potassium in mmol/L (median; IQR)	4.0 (3.7; 4.4)	3.9 (3.8; 4.2)	4.0 (3.7; 4.3)	-	0.637 †
Phosphorus in mmol/L (median; IQR)	1.15 (0.99; 1.27)	1.12 (0.97; 1.22)	1.12 (0.98; 1.23)	51	0.532 †
Total protein in g/L (median; IQR)	67 (63; 73)	65 (58; 72)	65 (60; 72)	17	0.472 †
Uric acid in mg/dL (median; IQR)	4.3 (2.9; 5.3)	4.3 (3.0; 5.8)	4.3 (3.0; 5.5)	19	0.650 †
Length of hospital stay (days) (median; IQR)	15 (11; 20)	10 (8; 15)	10 (9; 17)	-	0.002 *†
Intensive care unit	0 (0; 0)	0 (0; 0)	0 (0; 0)	-	0.759 †
Surgical ward	14 (10; 20)	10 (8; 12.5)	10 (8; 15)	-	0.005 *†
Surgical revision	5 (21.7%)	9 (11.1%)	14 (13.5%)	-	0.188 §
Anastomotic leak	1 (4.3%)	6 (7.4%)	7 (6.7%)	-	1.00 ¥
Mechanical ileus	3 (13.0%)	0 (0%)	3 (2.9%)	-	0.010 *¥
Paralytic ileus	5 (21.7%)	2 (2.5%)	7 (6.7%)	-	0.006 *¥
Postoperative bleeding	0 (0%)	3 (3.7%)	3 (2.9%)	-	1.00 ¥
Intraabdominal abscess	4 (17.4%)	4 (4.9%)	8 (7.7%)	-	0.072 ¥
Dehiscence of fascia	0 (0%)	2 (2.5%)	2 (1.9%)	-	1.00 ¥
Surgical site infection	1 (4.3%)	5 (6.2%)	6 (5.8%)	-	1.00 ¥
Most severe postoperative complication Clavien–Dindo				-	<0.001 *§
0	0 (0%)	40 (49.4%)	40 (38.5%)		
1–2	14 (60.9%)	20 (24.7%)	34 (32.7%)		
3–5	9 (39.1%)	21 (25.9%)	30 (28.8%)		

This table shows the initial 104 enrolled patients. Data were *n* (%) or median (25%; 75% interquartile range); HOS, high-output ileostomy; POD, postoperative day; §, chi-square test; †, Mann–Whitney U test; ¥, Fisher’s exact test; * *p* ≤ 0.05. The quantity of ileostomy output was documented per 24 h in millilitres; then, all the output that was documented during the hospital stay was added and a mean ileostomy output per day was calculated.

**Table 4:** Characteristics of patients with HOS during completed follow-up (defined as post-discharge HOS).

	HOS (*n* = 13)	No HOS (*n* = 90)	Total (*n* = 103)	Missing	*p* value
Follow up (days) (median; IQR)	118 (89; 141)	105 (80; 143)	105 (82; 142)	-	0.835 †
Age (years) (median; IQR)	62 (54.5; 66)	46 (28.8; 59)	48 (33; 62)	-	0.003 *†
Sex				-	0.190 §
Female	8 (61.5%)	38 (42.2%)	46 (44.7%)		
Male	5 (38.5%)	52 (57.8%)	57 (55.3%)		
Underlying diagnosis				-	0.090 §
Crohn’s disease	0 (0%)	29 (32.2%)	29 (28.2%)		
Ulcerative colitis	3 (23.1%)	20 (22.2%)	23 (22.3%)		
Ulcerative colitis-associated colorectal carcinoma	1 (7.7%)	8 (8.9%)	9 (8.7%)		
Colorectal carcinoma	6 (46.2%)	26 (28.9%)	32 (31.1%)		
Miscellaneous	3 (23.1%)	7 (7.8%)	10 (9.7%)		
HOS during hospital stay	0	23		-	
Readmission to hospital	7 (53.8%)	19 (21.1%)	26 (25.2%)	-	0.011 *§
Due to kidney injury	5 (38.5%)	2 (2.2%)	7 (6.8%)	-	<0.001 *§
Length of hospital stay including readmission (30 days postoperatively) (median; IQR)	10 (8; 13)	11 (9; 17)	11 (9; 17)	-	0.479 †
Most severe postoperative complication Clavien–Dindo				-	0.006 *§
1–2	10 (76.9%)	37 (41.1%)	47 (45.6%)		
3–5	3 (23.1%)	11 (12.2%)	14 (13.6%)		
Serum creatinine at follow-up in mg/dL (median; IQR)	1.04 (0.69; 1.28)	0.87 (0.75; 1.02)	0.88 (0.75; 1.04)	11	0.372 †
Acute kidney injury according to KDIGO	6 (46.2%)	5 (5.6%)	11 (10.7%)	-	<0.001 *§
Stage 1	2 (33.3%)	2 (40.0%)	4 (36.4%)		
Stage 2	1 (16.7%)	2 (40.0%)	3 (27.3%)		
Stage 3	3 (50.0%)	1 (20.0%)	4 (36.4%)		
Oral fluid intake (L/day) (median; IQR)	1.8 (1.5; 2.1)	2.0 (1.5; 2.0)	2.0 (1.5; 2.0)	-	0.777 †
Need for parenteral fluid	7 (53.8%)	12 (13.3%)	19 (18.4%)	-	<0.001 *§
Need for antidiarrheal medication	11 (84.6%)	34 (37.8%)	45 (43.7%)	-	0.001 *§
BMI at baseline in (kg/m^2^) (median; IQR)	24.7 (21.2; 32.5)	23.1 (19.9; 26.9)	23.9 (20.3; 27.2)	-	0.187 †
BMI at follow-up in (kg/m^2^) (median; IQR)	24.3 (19.8; 28.7)	23.3 (20.1; 26.2)	23.4 (20.0; 26.3)	-	0.762 †
Weight loss during follow-up	10 (76.9%)	32 (35.6%)	42 (40.8%)	-	0.005 *§
Weight loss in kg	2 (0.5; 12)	0 (0; 4)	0 (0; 4)	-	0.008 *†
Albumin in g/L (median; IQR)	41.5 (35.5; 49.0)	41.2 (38.5; 45.3)	41.2 (38.1; 45.9)	18	0.965 †
Magnesium in mmol/L (median; IQR)	0.84 (0.78; 0.87)	0.83 (0.79; 0.88)	0.83 (0.79; 0.88)	23	0.681 †
Calcium corrected in mmol/L (median; IQR)	2.31 (2.23; 2.48)	2.32 (2.25; 2.40)	2.32 (2.25; 2.40)	19	0.934 †
Sodium in mmol/L (median; IQR)	139 (136; 139)	139 (137; 141)	139 (137; 141)	11	0.234 †
Potassium in mmol/L (median; IQR)	4.1 (3.7; 4.5)	4.1 (3.8; 4.3)	4.1 (3.8; 4.3)	13	0.811 †
Phosphorus in mmol/L (median; IQR)	1.16 (0.91; 1.29)	1.13 (0.97; 1.27)	1.13 (0.97; 1.27)	33	0.884 †
Total protein in g/L (median; IQR)	71 (65; 79)	75 (71; 79)	75 (70; 79)	27	0.218 †
Uric acid in mg/dL (median; IQR)	5.5 (4.5; 7.3)	5.3 (4.5; 6.3)	5.4 (4.5; 6.5)	23	0.430 †
MUST ≥ 2	4 (30.8%)	21 (23.3%)	25 (24.3%)	-	0.559 §

This table shows the 103 patients who completed follow-up. Data were *n* (%) or median (25%; 75% interquartile range); IQR interquartile range; HOS, high-output ileostomy; POD, postoperative day; §, chi-square test; †, Mann–Whitney U test; * *p* ≤ 0.05; MUST, Malnutrition Universal Screening Tool; KDIGO, Kidney Disease: Improving Global Outcomes.

## Data Availability

The summarized data supporting the findings are included in the article. De-identified participant-level data are not publicly available because of the conditions of ethical approval and data-protection regulations. Additional data are available from the corresponding author on reasonable request.

## Abbreviations

AKI	Acute kidney injury
ASA	American Society of Anesthesiologists classification
BMI	Body mass index
FAP	Familial adenomatous polyposis
g/L	Grams per litre
HOS	High-output ileostomy
IBD	Inflammatory bowel disease
IQR	Interquartile range
KDIGO	Kidney Disease: Improving Global Outcomes
mg/dL	Milligrams per deciliter
mL	Millilitres
mmol/L	Millimoles per litre
MUST	Malnutrition Universal Screening Tool
